# Synthetic Incoherence via Scanned Gaussian Beams

**DOI:** 10.6028/jres.111.033

**Published:** 2006-12-01

**Authors:** Zachary H. Levine

**Affiliations:** National Institute of Standards and Technology, Gaithersburg, MD 20899-8410

**Keywords:** electron microscopy, Gaussian beams, incoherence, synthetic incoherence

## Abstract

Tomography, in most formulations, requires an incoherent signal. For a conventional transmission electron microscope, the coherence of the beam often results in diffraction effects that limit the ability to perform a 3D reconstruction from a tilt series with conventional tomographic reconstruction algorithms. In this paper, an analytic solution is given to a scanned Gaussian beam, which reduces the beam coherence to be effectively incoherent for medium-size (of order 100 voxels thick) tomographic applications. The scanned Gaussian beam leads to more incoherence than hollow-cone illumination.

## 1. Introduction

To perform microtomography, the first question is whether to use a coherent technique such as holotomography [1], or an incoherent technique, which ties the signals to mainstream tomography [2]. Amorphous samples are usually regarded as having incoherent interactions even with coherent beams [3], although coherent techniques have been applied to amorphous samples [4]. In biology, the samples are intrinsically amorphous if the growth of ice crystals is avoided. The now-standard technique of plunge freezing inhibits the formation of ice crystals and allows the preparation of amorphous samples. Tremendous progress has been made [5].

If the sample is crystalline or contains a significant proportion of crystallites, the incoherence must come from elsewhere. A suitable detector may observe an incoherent signal, as in the High-Angular Aperture Dark Field (HAADF) method, which exploits the fact that random motion of the nuclei in a crystal lead to an incoherent signal at large scattering angles [6]. (In the theoretical description, the Debye-Waller factors become very small.) Recently, the complement of the ideal HAADF signal has been used, under the name of Incoherent Bright Field (IBF) imaging [7] with the additional advantage that the signal is a monotonic function of thickness whereas a practical HAADF detector (with an inner diameter and an outer diameter) has a single-peaked thickness-intensity relation.

In this paper, a different approach is taken. A proposal is made to scan the beam in a particular manner while simultaneously recording an image to create a signal that is effectively incoherent. I call this approach “synthetic incoherence” to distinguish it from the more common situation of incoherence induced by thermal fluctuations of the source [8]. Using the definition of coherence, it is shown that it is possible to reduce the coherence of a beam across a surface to a level which is negligible tomographically. This is accomplished by scanning an out-of-focus Gaussian beam across the sample using a position-varying dwell time that itself has a Gaussian distribution. Of course, an incoherent beam cannot be strictly parallel, and this lack of parallelism sets a limit for the thickness of the sample assuming that the projection assumption [2] is required for the reconstruction, i.e., assuming that the beam travels in a straight line.

The definition of the mutual coherence function is given in many sources, such as Spence [9];

$$\Gamma_{12}({\vec{r}}_{1},{\vec{r}}_{2},T)=\underset{\tau \to \infty }{\lim}\frac{1}{2\tau }\int_{-\tau }^{\tau }dt\psi *({\vec{r}}_{1},t)\psi ({\vec{r}}_{2},t+T)$$ (1)

where ${\vec{r}}_{1}$ and ${\vec{r}}_{2}$ are two positions in the optical field *ψ*, *t* is a time, and *T* is a time difference. Only the equal time case, i.e., *T* = 0, is of interest here [10]. The complex degree of coherence is defined as the normalized mutual coherence function

$$\gamma_{12}({\vec{r}}_{1},{\vec{r}}_{2})=\frac{\Gamma_{12}({\vec{r}}_{1},{\vec{r}}_{2})}{{\left[\Gamma_{11}({\vec{r}}_{1})\Gamma_{22}({\vec{r}}_{2})\right]}^{1/2}}.$$ (2)

The normalized mutual coherence function is an intensity-weighted average of the phase factor between two illuminated points. When |*γ*_12_| ≪ 1 the beam is incoherent. Of course, $\gamma_{12}(\vec{r},\vec{r})=1$, so the best one can do is to have $\left|\gamma_{12}({\vec{r}}_{1},{\vec{r}}_{2})\right|$ go to zero rapidly as $\left|{\vec{r}}_{1}-{\vec{r}}_{2}\right|$ grows.

## 2. Gaussian-Scanned Gaussian Beams

The Gaussian wave function solution to the paraxial ray approximation for a beam traveling with its center in the *z* direction is [11]

$$\psi (x,y,z)=\frac{1}{1+iZ}\exp\left(-\frac{X^{2}+Y^{2}}{1+iZ}\right)$$ (3)

where the scaled dimensions *X*, *Y*, and *Z* are related to the physical dimensions *x*, *y*, and *z* by

$$X=\frac{x}{r_{0}}$$ (4)

$$Y=\frac{y}{r_{0}}$$ (5)

$$Z=\frac{\lambda z}{\pi r_{0}^{2}}$$ (6)

where *r*_0_ is a parameter characteristic of the beam waist corresponding to two standard deviations of the intensity, i.e., the square of the wave function and *λ* is the wavelength. The beam waist may be given in terms of the angle *θ*_1_ between the direction of maximum intensity and the angle at which the intensity has fallen by a factor of *e*^2^

$$r_{0}=\frac{\lambda }{\pi \theta_{1}}.$$ (7)

The time average of Eq. (2) may be performed by varying the beam conditions in a controlled fashion. This is in contrast to the usual practice of performing the time average of a source subject to statistical fluctuations. An analytic solution is achieved in the following case. The beam is scanned in the focal plane defined by *z* = 0. The beam center is given by (*x*_0_, *y*_0_). We restrict the discussion to the plane of a surface of the sample defined by the coordinate *z*, i.e., a plane whose normal is parallel to the beam direction. The mutual coherence function in the plane is given by

$$\begin{array}{l} \Gamma_{12}(x_{1},y_{1},z,x_{2},y_{2},z)=\int_{-\infty }^{\infty }\int_{-\infty }^{\infty }dX_{0}dY_{0}\frac{1}{1+Z^{2}}\\ \exp\left(-\frac{{\left(X_{1}-X_{0}\right)}^{2}+{\left(Y_{1}-Y_{0}\right)}^{2}}{1-iZ}-\frac{{\left(X_{2}-X_{0}\right)}^{2}+{\left(Y_{2}-Y_{0}\right)}^{2}}{1+iZ}\right)w(X_{0},Y_{0}) \end{array}$$ (8)

where *w*(*X*_0_, *Y*_0_) is a weighing factor which may be taken as proportional to the dwell time. The normalization

$$1=\int_{-\infty }^{\infty }dX_{0}dY_{0}w(X_{0},Y_{0})$$ (9)

is imposed. Assume that *w*(*X*_0_, *Y*_0_) = *w*(*X*_0_)*w*(*Y*_0_), i.e., *w* is a function of two variables factorized into two functions of one variable (which happen to be the same). The one-dimensional normalization

$$1=\int_{-\infty }^{\infty }dX_{0}w(X_{0})$$ (10)

is imposed, which implies the two-dimensional normalization of Eq. (9).

The function

$$\begin{array}{l} f(X_{1},X_{2})\\ =\int_{-\infty }^{\infty }dX_{0}\exp\left(-\frac{{\left(X_{1}-X_{0}\right)}^{2}}{1-iZ}-\frac{{\left(X_{2}-X_{0}\right)}^{2}}{1+iZ}\right)w(X_{0}) \end{array}$$ (11)

has an integrand that may be expressed as

$$\begin{array}{l} f_{I}=\exp\left(-\frac{X_{1}^{2}+X_{2}^{2}-2(X_{1}+X_{2})X_{0}+2X_{0}^{2}}{1+Z^{2}}\right)\\ \exp\left(i\frac{Z}{1+Z^{2}}[-X_{1}^{2}+X_{2}^{2}+2(X_{1}-X_{2})X_{0}]\right)w(X_{0}). \end{array}$$ (12)

The factor not dependent on *X*_0_ is

$$C=\exp\left(-\frac{X_{1}^{2}+X_{2}^{2}}{1+Z^{2}}\right)\exp\left(i\frac{Z}{1+Z^{2}}(-X_{1}^{2}+X_{2}^{2})\right).$$ (13)

Specialize the weight to a Gaussian

$$w(X_{0})=\frac{1}{\sqrt{2\pi }\Sigma }\exp\left(-\frac{X_{0}^{2}}{2\Sigma^{2}}\right)$$ (14)

where Σ = *σ*/*r*_0_ is the scaled standard deviation and *σ* is the dimensional standard deviation. For this weight function

$$\begin{array}{l} f(X_{1},X_{2})=\frac{C}{\sqrt{2\pi }\Sigma }\int_{-\infty }^{\infty }dX_{0}\exp(-\alpha X_{0}^{2})\\ \exp\left(\frac{-2(X_{1}+X_{2})+2iZ(X_{1}-X_{2})}{1+Z^{2}}X_{0}\right) \end{array}$$ (15)

where *α* = 2(1 + *Z*^2^)^−1^ + (2Σ^2^)^−1^. This integral may be performed analytically using the result

$$\int_{-\infty }^{\infty }dx\exp(-ax^{2}+bx)=\sqrt{\frac{\pi }{a}}\exp\left(\frac{b^{2}}{4a}\right).$$ (16)

So

$$\begin{array}{l} f(X_{1},X_{2})=\frac{C}{\sqrt{2\alpha }\Sigma }\exp\left(\frac{{\left(X_{1}+X_{2}\right)}^{2}-Z^{2}{\left(X_{1}-X_{2}\right)}^{2}}{\alpha {\left(1+Z^{2}\right)}^{2}}\right)\\ \exp\left(-2i\frac{Z(X_{1}^{2}-X_{2}^{2})}{\alpha {\left(1+Z^{2}\right)}^{2}}\right). \end{array}$$ (17)

The 1D normalization constant may be obtained by setting *X*_2_ to *X*_1_ in Eq. (17), then returning to Eq. (17) and setting *X*_1_ to *X*_2_, multiplying, and taking the square root. The result is

$$f^{norm}=\frac{C}{\sqrt{2\alpha }\Sigma }\exp\left(\frac{2(X_{1}^{2}+X_{2}^{2})}{\alpha {\left(1+Z^{2}\right)}^{2}}\right).$$ (18)

The quotient simplifies to

$$\frac{f(X_{1},X_{2})}{f^{norm}}=\exp\left(-\frac{{\left(X_{1}-X_{2}\right)}^{2}}{\alpha \left(1+Z^{2}\right)}\right)\exp\left(-2i\frac{Z(X_{1}^{2}-X_{2}^{2})}{\alpha {\left(1+Z^{2}\right)}^{2}}\right).$$ (19)

Note that $\gamma_{12}({\vec{r}}_{1},{\vec{r}}_{2})$ at *z*_1_ = *z*_2_ = *z* is simply Eq. (19) times Eq. (19) under the substitution (*X*_1_, *X*_2_) → (*Y*_1_, *Y*_2_). Let *R*^2^ = (*X*_1_−*X*_2_)^2^ + (*Y*_1_ – *Y*_2_)^2^. Finally,

$$|\gamma_{12}|=\exp\left(-\frac{R^{2}}{\alpha (1+Z^{2})}\right)=\exp\left(-\frac{2\Sigma^{2}R^{2}}{4\Sigma^{2}+1+Z^{2}}\right).$$ (20)

Note that |*γ*_12_| depends on the coordinates only through the difference variable *R*. Eq. (20) obeys *γ*_12_ = 1 for *R* = 0, as required. If Σ → ∞, |*γ*_12_| = exp(−*R*^2^/2). This means that if the beam is scanned uniformly over the entire (infinite) focal plane, the range of the mutual coherence is limited to about the focal spot size. (*R* is the distance between any two points in the plane in units of the focal spot size parameter *r*_0_.) On the other hand, if there is no scanning, i.e., if Σ → 0, then |*γ*_12_| = 1 confirming that a single Gaussian is perfectly coherent.

How narrow can the mutual coherence function be made in practice? The intermediate cases may be parameterized by a constant *c*_1_ defined by $c_{1}^{2}=(1+Z^{2})/\Sigma^{2}$, in which case the coherence scale length is $\sqrt{1+c_{1}^{2}/4}$ times the asymptotic limit. The case $c_{1}^{2}=12$, for example, is a reasonable compromise which achieves a moderate scan range and for which the scale length of the normalized mutual coherence function is twice its asymptotic minimum. To simplify the analysis, assume *Z* ≫ 1, which will be true for typical TEM illumination. Then we may approximate *c*_1_Σ = *Z* or

$$\sigma =\frac{\lambda z}{c_{1}\pi r_{0}}=\frac{z\theta_{1}}{c_{1}}$$ (21)

in the unscaled variables. Now *zθ*_1_ is the characteristic scale length of the illuminated region, which is about the sample size. Because we have a Gaussian weighting function, we make a very small error by omitting the tails. In practice, the scanning must take place over a finite range, which we may call *c*_2_*σ*. The case *c*_2_ = 6 represents the omission of the tails past 3 standard deviations; the total scanned length is $\sqrt{3}z\theta_{1}$ in this case, i.e., roughly twice the sample size, which is a practical result.

The situation imposes a limit for projection tomography. A typical figure for *θ*_1_ is 3 mrad. Hence, the illumination strikes the sample by rays which are going in different directions on this order. If the results are to be applied in projection tomography, the number of pixels must be below some constant or order 1 over *θ*_1_, which means below some constant times 333 for the case of *θ*_1_ = 3 mrad. In practice, tomographic reconstructions are performed over 100 to 1000 voxels, so the requirement is consistent with smaller reconstructions but not larger ones.

## 3. Hollow-Cone Illumination

**Fig. 1:**
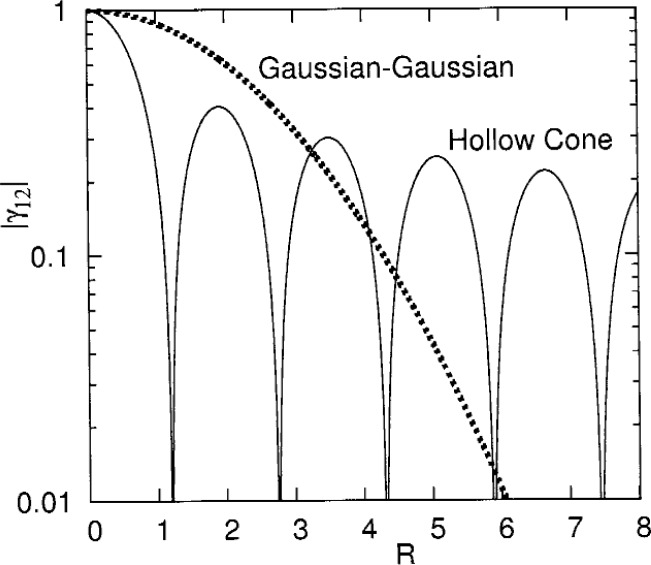
The mutual coherence function vs. the distance between the two points in the plane *R* = [(*x*_2_ – *x*_1_)^2^ + (*y*_2_ – *y*_1_)^2^]^1/2^/*r*_0_ for the Gaussian beam scanned with a Gaussian intensity profile (dotted line) and hollow-cone illumination (solid line) with $c_{1}^{2}=12$, *θ* = 3 mrad, *λ* = 1.969 pm, *z* = 1 mm. These parameters yield *r*_0_ = 209 pm. The parameters are defined in the text.

Hollow-cone illumination is frequently used to enhance the incoherence of the beam [12]. Several electron microscope vendors allow such a mode in hardware. The analysis of hollow-cone illumination is deferred to a future paper [13]; here, a numerical example is provided. In hollow-cone illumination with a Gaussian beam, the axis of the fundamental solution given in Eq. (3) is tipped by the polar angle *θ* and azimuthal angle *ϕ*. The polar angle *θ* is held constant and the azimuthal angle *ϕ* is varied over its full range of 0 to 2π. An example of the resulting mutual coherence function is given in Fig. 1 for a 300 keV electron with *θ* = 3 mrad, hence a wavelength *λ* = 1.969 pm, and *z* = 1 mm. In the example, the focus of the beam remains at the same point in space as the azimuthal angle *ϕ* is varied. The situation of this example is characteristic: the loss of confinement appears to have an envelope of 1/*R*—a far slower decay than a Gaussian beam scanned with a Gaussian intensity as discussed in the previous section and also as shown in the figure.

## 4. Conclusions

To achieve a practically incoherent beam for use in electron tomography, a novel illumination scheme is proposed that combines elements of STEM and TEM: the sample is to be completely illuminated throughout the observation while the beam is scanned. Moreover, the scanning itself is more concentrated near the center so that the intensity is given by a Gaussian with a standard deviation under user control. The new scheme is compared to hollow-cone illumination and holds promise for removing the residual long-range correlations in the latter case. The new scheme leads to no substantial correlations for separations of 1 nm or more in an example chosen with realistic parameters.

The practical implementation of the scheme requires the experimentalists to be able to program the controls of a STEM-TEM. Such a group would probably involve a collaboration with a vendor or the use of one of the limited number of electron microscopes dedicated to research into the techniques of microscopy.
